# An Unusual Case of Two Paraneoplastic Neurological Syndromes in a Patient With Lung Cancer

**DOI:** 10.7759/cureus.33047

**Published:** 2022-12-28

**Authors:** Shermila Pia, David Looi, Robert Stone, Ning Zhong, Forshing Lui

**Affiliations:** 1 Neurology, University of Rochester, Rochester, USA; 2 Neurology, California Northstate University College of Medicine, Elk Grove, USA; 3 Neurology, Kaiser Permanente Sacramento Medical Center, Sacramento, USA; 4 Clinical Sciences, California Northstate University College of Medicine, Elk Grove, USA

**Keywords:** crmp5, gabab, surface/synaptic, intracellular, paraneoplstic

## Abstract

Paraneoplastic immune-mediated disorders have been well described in the literature. However, it is still relatively rare. The incidence has increased over the past decade due largely to the discovery of more autoantibodies. With a better understanding of the pathophysiology of different autoantibodies and clinical phenotypes, we are often able to diagnose clinically some specific paraneoplastic autoimmune neurological syndromes. We may also predict the response to treatment based on the autoantibody class. We are presenting a very unusual case of two completely different paraneoplastic syndromes with two different autoantibodies, gamma-aminobutyric acid-B (GABA_B_) and collapsin response mediator protein 5 (CRMP5), in a patient with underlying small-cell lung cancer. We will discuss the differences in the two antibody syndromes, their significance, and their management.

## Introduction

Paraneoplastic nervous system disorders are neurological disorders occurring as an indirect, often immune-mediated response to underlying cancer rather than direct invasion by the cancer cells. They are relatively rare, with an incidence of 0.89 per 100,000 person-years, a prevalence of 4.37 per 100,000, and roughly one in every 300 cancers [1]. Over the past decade, the incidence and prevalence have increased mostly due to the identification of more autoantibodies [2].

With the discovery of more autoantibodies associated with underlying cancer, we are able to understand more about the pathophysiology of immune-mediated paraneoplastic neurological syndromes [3-6]. The first group of paraneoplastic syndromes is associated with intracellular antigens. These are autoantibodies such as antineuronal nuclear antibodies type 1 (ANNA-1 or anti-Hu antibody) and type 2 (ANNA-2 or anti-Ri antibody). These autoantibodies are not directly pathogenic, and immune therapy is generally ineffective. The newer identified group is those antibodies targeting cell surface or synapses, such as anti-N-methyl-D-aspartate (NMDA) receptor antibodies. These can present as paraneoplastic syndromes or simply idiopathic autoimmune diseases, which generally respond well to immunotherapy.

We present a very rare case of a combination of two paraneoplastic syndromes in a patient with small-cell lung cancer. We will discuss the clinical presentation, autoantibodies, pathophysiology, and management in more detail.

## Case presentation

A 66-year-old woman with a 50-pack-year smoking history experienced an episode of left-sided visual disturbance confirmed to be optic neuritis two years before her presentation to our service. She went on to develop impaired balance, upper and lower extremity weakness, Lhermitte’s sign, urinary incontinence, burning paresthesia, and anesthesia of the S2-4 dermatomes. Her symptoms were progressive, and she became wheelchair dependent with minimal vision in her left eye. Lumbar puncture reviewed oligoclonal bands in her cerebrospinal fluid (CSF). The aquaporin-4 (AQP4) antibody was negative. Magnetic resonance imaging (MRI) brain showed an increased signal in the left optic nerve and no other abnormalities. MRI spine did not show any lesions. She was diagnosed with probable multiple sclerosis.

One year before the presentation, she was diagnosed with small-cell lung cancer (SCLC), for which she received chemotherapy and prophylactic whole-brain radiation.

At the time of her presentation to us, she had two months of new-onset intractable seizures and worsening short-term memory. Her seizures consisted of spells of unresponsiveness with postictal confusion and were refractory to two antiepileptic medications at adequate doses.

The patient’s examination was notable for short-term memory and cognitive deficits (Montreal Cognitive Assessment test (MOCA) 19/30 with 0/5 delayed recall). She had left optic disc pallor, a left afferent pupillary defect, and reduced visual acuity (20/40 on the right and 20/100 on the left). She was non-ambulatory, with 3/5 strength in the left leg and 4/5 in the right leg. Sensory testing revealed S2-4 anesthesia. Reflexes were 2+ throughout, with upgoing plantar responses.

**Figure 1:**
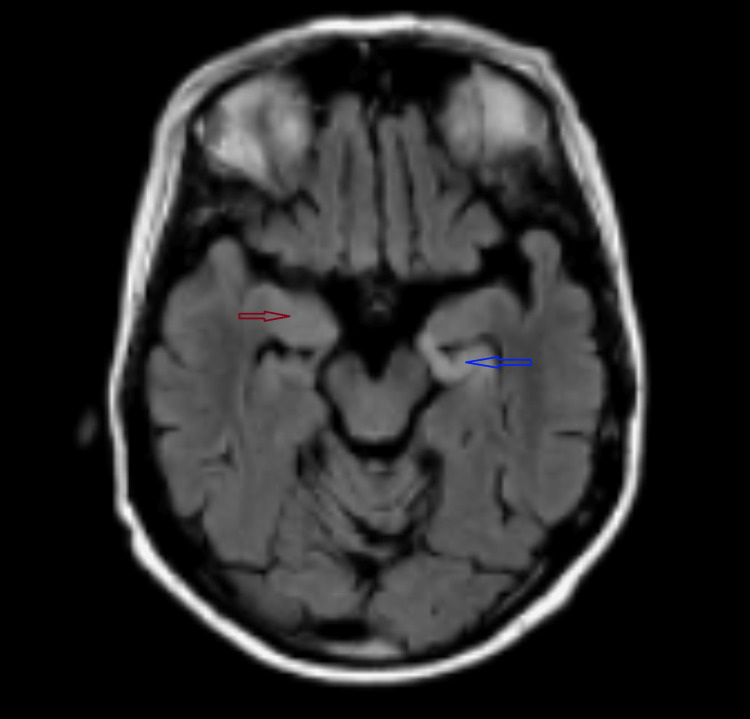
MRI brain - FLAIR sequence MRI of the brain shows T2/fluid attenuated inversion recovery (FLAIR) hyperintensity throughout the left hippocampus (blue arrow) and right amygdala (red arrow) with atrophy of the right hippocampus. There are no contrast-enhancing lesions.

She had a normal interictal electroencephalogram (EEG). CSF showed the presence of oligoclonal bands and elevated IgG index with normal cell counts, protein, culture, and encephalitis panel. Aquaporin4 (AQP4 IgG) and anti-myelin-oligodendrocyte glycoprotein (MOG IgG) testing were negative. MRI showed T2 hyperintensity throughout the left hippocampus and right amygdala with atrophy of the right hippocampus; there were no contrast-enhancing lesions (Figure 1).

A serum autoimmune encephalopathy panel was positive for both gamma-aminobutyric acid-B (GABA_B_) and collapsin response mediator protein 5 (CRMP5) antibodies at high titers. The patient was diagnosed with autoimmune limbic encephalitis secondary to anti-GABA_B_ receptor antibodies. Her myelitis and optic neuritis were determined to be paraneoplastic in the setting of known SCLC and anti-CRMP5 (also called anti-CV2) antibodies. The patient was started on treatment with rituximab and subsequently showed significant improvement in her seizure control. However, her sensorimotor deficits remained, and she continued to have cognitive decline and occasional breakthrough seizures.

## Discussion

Autoimmune encephalitides and their related neurological disorders are increasingly recognized for their association with specific neuronal autoantibodies [3-6]. These disorders may affect any area of the brain, but most commonly, the limbic structures or medial temporal lobes [4,5]. CSF studies can show signs of inflammatory change, such as pleocytosis, elevated protein, and oligoclonal bands [7]. Antibodies may or may not be associated with an underlying malignancy. Clinically, paraneoplastic syndromes should be suspected in patients with subacute, progressive neurologic symptoms and existing or high risk for malignancy (e.g., smokers). There are two main classes of autoantibodies, intracellular (target an antigen in the nucleus or cytoplasm) and cell-surface/synaptic (target an antigen in the neuronal synapse or on the cell membrane).

Intracellular antibodies are considered secondary antibodies since they form against antigens in the nuclear or cytoplasmic epitopes after a separate disease process has already damaged the neuron. Intracellular antibodies have not yet been proven to play a direct pathogenic role in mediating disease, and their presence is used instead as a marker of disease. There is some evidence that these diseases are mediated mainly through a predominant T-cell-mediated mechanism with heavy involvement of CD4+ and CD8+ T cells [8]. However, further investigation of intracellular antibodies’ pathophysiology and contribution to the neurologic disease process is needed. The vast majority of intracellular antibodies are paraneoplastic; therefore, they are also called ‘onconeural’ antibodies. Neurologic symptoms frequently precede the discovery of the malignancy in weeks, months, or even years [4-6]. Immunosuppression for diseases involving intracellular antibodies has not proven to be significantly effective [5]. Treatment is typically focused on the underlying malignancy.

On the other hand, cell surface/synaptic antibodies are frequently directly pathogenic. These antibodies target functional antigens on neuronal surfaces (e.g., ion channels, receptors), leading to a B-cell-mediated immune response and inflammation. They are less consistently associated with underlying malignancy than intracellular antibodies. Treatment of cell surface antibody-mediated disease involves plasmapheresis or immunosuppression (e.g., corticosteroids, rituximab, intravenous immunoglobulin [IVIg]) in addition to treating any underlying tumors [9].

GABA_B_ receptor antibody limbic encephalitis

The GABA_B _receptor antibody is a cell surface antibody primarily known to cause limbic encephalitis and is associated with an underlying neoplasm in about half of cases [10]. The most common underlying tumor is SCLC, not other types of lung cancer [11]. Since its identification in 2010, GABA_B _receptor antibody encephalitis has remained an uncommon condition, with a few hundred cases identified to date [1]. Cases have been identified across a wide range of ages, including both pediatric and adult populations. Limbic encephalitis associated with GABA_B_ receptor antibodies often presents with subacute (days to months) seizures, cognitive decline, short-term memory deficits, and behavioral or psychiatric disturbance. Seizures in this form of limbic encephalitis are classically focal with impaired awareness and often resistant to standard anti-epileptic medications [10,12,13].

CRMP5 antibody-associated myelitis, optic neuritis

The CRMP5 antibody is an intracellular antibody that is almost always paraneoplastic and most commonly seen in SCLC (about 75% of cases), again not with other types of lung cancer [14,15]. Clinically, CRMP5 is frequently associated with painful peripheral neuropathies and cerebellar ataxia. It can also be associated with optic neuritis (in about 10% of patients) and myelitis (in about 20% of patients) [14,16]. The patient presented with symptoms of CRMP5 neuropathy (burning paresthesia, optic neuritis, and myelitis) several years before identifying the tumor and causative antibody, a delay common in intracellular antibody-associated conditions [1,4].

In the case of our patient, the combination of optic neuritis and presumed myelitis with CSF oligoclonal bands led to an initial diagnosis of probable multiple sclerosis (MS). Patients with atypical presentations of MS or neuromyelitis optica spectrum disorder (NMOSD) and significant risk factors for malignancy, autoimmune or paraneoplastic encephalitis should be considered early with a high degree of suspicion.

## Conclusions

The incidence and prevalence of autoimmune encephalitis have increased dramatically during the past decade due to the identification of novel autoantibodies. Many of these cases present as paraneoplastic syndromes, often preceding the final diagnosis of underlying cancer. Overall, these syndromes are still relatively rare. Of the two main types of autoantibodies, those against intracellular antigens are not directly pathogenic and instead serve as a disease marker for underlying malignancy. In contrast, autoantibodies against cell surface or synaptic antigens are directly pathogenic as they induce a B-cell-mediated autoimmune response.

Our patient is unusual due to the presence of both types of autoantibodies associated with underlying SCLC. The patient presented over a two-year period, first with CRMP5-associated paraneoplastic syndrome one year before the cancer diagnosis and later with a typical GABA_B_ paraneoplastic autoimmune limbic encephalitis after the cancer diagnosis. Our case also demonstrated the clinical features of the two paraneoplastic immune-mediated neurological disorders.
